# Preimplantation development analysis of aneuploid embryos with different chromosomal abnormalities

**DOI:** 10.1016/j.heliyon.2024.e40686

**Published:** 2024-11-26

**Authors:** Keyi Si, Bingxin Ma, Jian Bai, Li Wu, Hui He, Lei Jin, Bo Huang

**Affiliations:** Reproductive Medicine Center, Tongji Hospital, Tongji Medicine College, Huazhong University of Science and Technology, Wuhan, 430030, China

**Keywords:** Time-lapse, Aneuploidy, Confounding, Morphokinetic parameters, Chromosomal abnormalities, Next-generation sequencing

## Abstract

**Background:**

The change of morphokinetic pattern in aneuploid embryos will facilitate the non-invasive selection of euploid embryos. In this study, we investigated the impact of different chromosomal abnormalities on the morphokinetic patterns of embryonic development.

**Methods:**

Our cohort includes 939 time-lapse preimplantation genetic testing cycles performed between January 2019 and July 2022 at a single academic fertility center, with a total of 2876 biopsied blastocysts. Intracytoplasmic sperm injection, blastocyst culture, trophectoderm biopsy, time-lapse monitoring, and next-generation sequencing were performed.

**Results:**

After adjusting for patient- and cycle-related factors, **s**ix morphokinetic parameters (t5, P = 0.006; t8, P = 0.048; tSB, P < 0.001; tB,P < 0.001; t5-t2, P = 0.004; tB-tSB, P < 0.001) were significant in multilevel mixed-effects logistic regression model analysis for morphokinetic parameters to predict euploid or aneuploid embryos. None of the patient- or cycle-related factors systematically affected any morphokinetic parameter. Morphokinetic parameters of late cleavage and blastocyst stages in embryos with chromosome fragment deletion (t4 to t8, tB, t5-t2, tB-tSB, ECC2, ECC3, s2, P < 0.05) or duplication (t4, t5, tSB, tB, t5-t2, P < 0.05) were prolonged, and the morphokinetic parameters of the blastocyst stage in monosomic embryos (tSB, tB, tB-tSB, P < 0.01) were prolonged. Partial or complete chromosome 20 or 22 deletion can cause significant delays in multiple parameters of cleavage and blastocyst stages (from t4 to tB, P < 0.05).

**Conclusions:**

Our study found that different chromosomal abnormalities have different effects on the morphokinetic parameters. Significant delays in morphokinetic parameters at different stages were found in fragment-mutated embryos and monosomic embryos. This can provide insights into the pre-implantation development pattern of aneuploid embryos and help non-invasive embryo selection.

## 1 Introduction

Aneuploidy can disrupt mitosis, and interfere with cell-cell interactions and blastocyst differentiation, leading to changes in the cleavage behavior of embryos cultured in vitro [1], is one of the causes of implantation failure and miscarriage [2,3]. Preimplantation genetic testing (PGT) can accurately assess aneuploid and mosaic embryos before transfer by performing a biopsy on trophectoderm cells from blastocysts; however, its invasive nature limits its applicability in certain in vitro fertilization (IVF) scenarios [[4], [5], [6], [7]]. Time-lapse microscopy (TLM) allows real-time tracking and recording of embryo development, helping embryologists identify and evaluate transient morphological features and specific dynamic events in pre-implantation embryo development [[8], [9], [10]], including reverse cleavage, irregular divisions, and blastocyst collapse [[11], [12], [13]]. These events and features may be associated with aneuploidy [[14], [15], [16]].

A high rate of aneuploidy may be the main cause of early embryonic failure [17], but some aneuploid embryos exhibit morphological qualities similar to euploid embryos. Developmental speed may serve as an independent biomarker of embryo quality among embryos with morphological quality without obvious abnormalities. When controlling for female age, male age, number of previous embryo transfer cycles, inner cell mass and trophectoderm morphological grades, and progesterone supplementation, blastocysts formed on day 6 showed significantly lower implantation rates, clinical and ongoing pregnancy rates, and live birth rates compared to those formed on day 5. In embryos forming blastocysts between days 4 and 7, differences in the timing of developmental stages and related intervals became increasingly pronounced, already evident at the time of pronuclear fading [18]. Compared to blastocysts formed on day 5 or day 6, those formed on day 7 had a higher risk of aneuploidy [19,20].

Morphokinetic parameters can more precisely quantify the developmental speed at various stages of preimplantation embryos. Numerous studies have attempted to correlate morphokinetic parameters with embryo ploidy; however, the significant heterogeneity between different research designs and sample populations results in certain differences in the conclusions of these models [14]. Most previous studies do not account for the influence of clustering and confounding factors, which can lead to model overestimation [14,21]. Three studies used cluster data analysis [22,23] or sibling embryo analysis [24] to investigate the relationship between morphokinetic parameters and ploidy status. These studies take into account that all embryos from the same patient in the same cycle may be affected by the same patient- and cycle-related factors and their conclusions are relatively reliable.

Different types and severities of aneuploidy may have distinct effects on pre-implantation embryo development, reflected in variations in morphokinetic parameters [25]. Recent studies have found that inducing aneuploidy in mouse oocytes during meiosis significantly prolongs the timing of morphokinetic events, and this prolongation is proportional to the severity of aneuploidy [26]. Currently, there is limited research on the relationship between morphokinetic parameters and the types of chromosomal variations in aneuploid embryos, with two relevant studies reaching different conclusions [24,27]. Therefore, further research is needed to explore this issue in greater depth.

This study aims to use cluster analysis to control potential confounders, identify morphokinetic parameters that predict embryo ploidy, and analyze the characteristics of morphokinetic parameters associated with different types of chromosomal abnormalities. This study will assist embryologists in comprehensively evaluating the details of embryo development and aid in non-invasive ploidy prediction.

## 2 Methods and materials

### 2.1 Study design and participants

2876 biopsied blastocysts in 939 time lapsed-PGT cycles performed between January 2019 and July 2022 at the Reproductive Medicine Center, Huazhong University of Science and Technology Hospital were included in this study. Embryos were imaged using the Embryoscope Plus time-lapse microscopy system (Vitrolife, Denmark) from post-insemination to biopsy and cryopreservation. Institutional review board approval was not required because all patients in this study underwent routine clinical treatment and no additional interventions were provided to all patients.

### 2.2 ICSI, embryo incubation, and timelapse monitoring

The procedures for semen and cumulus-oocyte complex (COC) preparation, insemination, and embryo culture were performed according to our previous publication [28]. In brief, semen was obtained via masturbation and subsequently processed using density gradient centrifugation. The sperm concentration, motility, and morphology were assessed following the fifth edition of the World Health Organization guidelines. In ICSI cycles, COCs were denuded with a brief exposure to HYASE (Vitrolife, Sweden) containing hyaluronidase, before sperm injection into metaphase II (MII) oocytes. Zygotes were then transferred to G1 Plus (Vitrolife, Sweden) and cultured using an Embryoscope Plus time-lapse microscope system (Vitrolife, Denmark), which captured images of each embryo every 10 min. All fertilized oocytes were cultured at 37 °C under 6 % CO2, 5 % O2, and 89 % N2 until biopsy or cryopreservation on Day 5 or 6. For biopsy, a laser was used to create an opening in the zona pellucida, and 3–6 trophectoderm cells were collected through mechanical dissection. During the biopsy, a laser was used to create a small opening in the zona pellucida, and 3–6 trophectoderm cells were collected through mechanical dissection. The grading of blastocyst inner cell mass and trophectoderm was done according to the Gardner scoring system [29].

### 2.3 Time-lapse monitoring and definitions of morphokinetic parameters

The Embryoviewer software was employed for the examination of morphokinetic parameters in embryos that were cultured within the Embryoscope imaging system. These morphokinetic parameters were assessed manually, following the criteria outlined by Ref. [30]. These parameters encompassed various developmental milestones, including the mid-time of ICSI (t0), the appearance of two pronuclei (tPNa), the time of pronuclei disappearance (tPNf), the division of the embryo into two to eight discrete cells (t2-t8), the initiation of blastulation (tSB), the full blastocyst formation time (the last frame before the zona pellucida begins to thin) (tB), the second cell cycle (ECC2 = t4 – t2), the third cell cycle (ECC3 = t8 – t4), the synchronicity of the two blastomere divisions (s2 = t4 – t3), and the synchronicity of the four blastomere divisions (s3 = t8 – t5).

### 2.4 Next-generation sequencing and classification of ploidy

In all PGT cycles, the methodology of next-generation sequencing (NGS) was employed, and this approach has been elucidated in prior descriptions [28]. The threshold was above 70 % for aneuploidy detection. Lower euploidy limits were 30 % for chromosomes 13, 16, 18 and 21, 50 % for chromosome 19 and 40 % for others.

### 2.5 Statistical analysis

Statistical analyses were conducted utilizing the Statistical Package for Social Sciences, version 13.0 (SPSS). Continuous variables were presented as mean values with standard deviations (mean ± SD) and were compared through the Mann–Whitney *U* test. Categorical variables, on the other hand, were compared using Fisher's exact test or chi-squared tests.

To address the inherent correlation among observations within the same cluster, multilevel mixed-effects models were employed. Given that embryos generated by the same patient do not offer independent data, a multilevel random effects model with two levels was utilized. The first level represents individual embryos, and the second level represents the cycle from which they originated. This model was adjusted for potential confounding factors, including patient and cycle characteristics.

Statistical significance was determined at a threshold of P < 0.05.

## 3 Results

Table 1 summarizes the main descriptive features of the cycles in this study. The mean age of the patients was 31.9 ± 4.6 years. In 939 TLM-PGT cycles, a total of 2876 embryos were biopsied. Among them, 1260 (43.8 %) euploid embryos, 331 (11.5 %) mosaic embryos, and 1285 (44.7 %) aneuploid embryos were used for analysis.

**Table 1:** Clinical characteristics of PGT cycles.

Parameter	
No. of cycles	939
Total number of oocytes retrieved	14577
Total number of matured oocytes	11449
Total number of two pronucleus (2 PN)	8316
Total successfully amplified blastocyst	5491
Total blastocyst biopsied	2876
No. of euploidy	1260
No. of aneuploidy	331
No. of mosaicism	1285
Age (y)	31.9 ± 4.6
BMI (kg/m2)	22.3 ± 6.8
Duration of infertility (y)	2.5 ± 2.3
Level of FSH/100 (IU)	7.4 ± 2.6
Level of AMH/100 (IU)	4.7 ± 7.3
No. of retrieved oocytes per cycle	13.7 ± 7.9
Time of ovarian stimulation (days)	9.9 ± 1.8

BMI, Body Mass Index; FSH, Follicle-Stimulating Hormone; AMH, Anti-Müllerian Hormone.

### 3.1 Morphokinetic parameters of euploid, mosaic, and aneuploid embryos

**Fig. 1:**
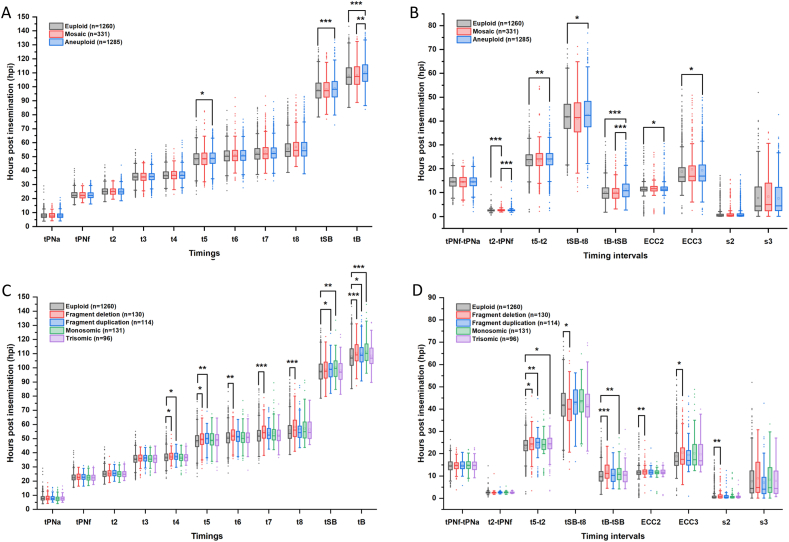
(a, b) Morphokinetic parameters of euploid, mosaic and aneuploid embryos. (c, d) Morphokinetic parameters of embryos with different chromosomal abnormalities. The groups with significant differences (P < 0.05) were marked. (∗, P < 0.05; ∗∗, P < 0.01; ∗∗∗, P < 0.001).

Due to the broad range of time-lapse events, euploid, mosaic, and aneuploid embryos overlapped in almost all morphokinetic parameters (Fig. 1A and B; Supplementary Table 1). Univariate analysis showed that aneuploid embryos had significant delays in t5 (P = 0.022), tSB (P = 0.001), tB (P < 0.001), t5-t2 (P = 0.006), tSB-t8 (P = 0.043), tB-tSB (P < 0.001), ECC2 (P = 0.036), and ECC3 (P = 0.046) compared with euploid embryos, and in tB (P = 0.002), and tB-tSB (P < 0.001) compared with mosaic embryos. Mosaic embryos had significantly prolonged t2-tPNf compared with both euploid and aneuploid embryos (P < 0.001). Notably, tB was progressively delayed in euploid, mosaic, and aneuploid embryos (107.9 ± 8.8, 108.4 ± 9.1, 110.2 ± 8.9, respectively; P < 0.05).

Multilevel mixed-effects logistic regression model assessed the relationship between TLM parameters and patient- or cycle-related characteristics (age, body mass index (BMI), and infertility duration for each female patient; the number of retrieved oocytes, level of follicle-stimulating hormone (FSH), level of anti-müllerian hormone (AMH), and time of ovarian stimulation for each cycle; see Supplementary Table 2). No single factor systematically affects any TLM parameter. BMI (t2-tPNf, tB – tSB), infertility duration (tB-tSB), level of FSH (tB-tSB, ECC3), and time of ovarian stimulation for each cycle (tSB, tB, tB-tSB) had significant influences on specific parameters (P < 0.05). Among the TLM parameters, tB-tSB was most influenced by patient- or cycle-related factors. In a multilevel mixed-effects logistic regression predicting embryo ploidy (euploid or aneuploid) using TLM parameters, these factors were considered as potential confounders. After adjustment, six TLM parameters (t5, P = 0.006; t8, P = 0.048; tSB, P < 0.001; tB,P < 0.001; t5-t2, P = 0.004; tB-tSB, P < 0.001) remained statistically significant (Table 2).

**Table 2:** Multilevel mixed-effects logistic regression model analysis for morphokinetic parameters and patient and cycle characteristics to predict the ploidy status of embryos.

Morphokinetic parameters	OR (95 % CI)	P value
tPNa (hpi) (n = 2508)	1.013 (0.971–1.056)	0.546
tPNf (hpi) (n = 2511)	0.998 (−0.966 to 1.031)	0.910
t2 (hpi) (n = 2516)	0.995 (0.965–1.027)	0.760
t3 (hpi) (n = 2418)	0.989 (0.968–1.010)	0.299
t4 (hpi) (n = 2484)	0.989 (0.968–1.011)	0.325
t5 (hpi) (n = 2434)	0.982 (0.969–0.995)	0.006
t6 (hpi) (n = 2234)	0.990 (0.975–1.005)	0.192
t7 (hpi) (n = 2226)	0.998 (0.974–1.002)	0.104
t8 (hpi) (n = 2297)	0.989 (0.978–1.000)	0.048
tSB (hpi) (n = 2389)	0.981 (0.971–0.992)	<0.001
tB (hpi) (n = 2382)	0.968 (0.958–0.978)	<0.001
tPNf-tPNa (h) (n = 2497)	0.990 (0.959–1.023)	0.553
t2-tPNf (h) (n = 2501)	0.9939 (0.811–1.088)	0.450
t5-t2 (h) (n = 2419)	0.978 (0.963–0.993)	0.004
tSB-t8 (h) (n = 2180)	0.990 (0.980–1.001)	0.067
tB-tSB (h) (n = 2377)	0.928 (0.907–0.950)	<0.001
ECC2 (h) (n = 2471)	0.978 (0.944–1.012)	0.206
ECC3 (h) (n = 2282)	0.988 (0.975–1.002)	0.092
s2 (h) (n = 2397)	1.010 (0.976–1.048)	0.538
s3 (h) (n = 2265)	1.001 (0.989–1.014)	0.837

### 3.2 Morphokinetic parameters of different ploidy statuses

We explored whether the type of aneuploidy affected the TLM parameters (Fig. 1C and D; Supplementary Table 3). Aneuploid blastocysts are reclassified into 4 subgroups according to their type of chromosome abnormalities: the fragment deletion group with subchromosomal deletion of segmental nature only (n = 130), the fragment duplication group with subchromosomal duplication of segmental nature only (n = 114), the monosomic group with one whole chromosome missing (n = 131), and the trisomic group with one whole chromosome repeating (n = 96). Compared to euploid embryos, the fragment deletion group showed significant prolongation in late cleavage (t4 to t8, t5-t2, ECC2, ECC3, s2) and blastocyst stage (tB, tB-tSB) parameters, and the fragment duplication group had prolonged late cleavage (t4, t5, t5-t2) and blastocyst (tSB, tB) parameters (P < 0.05). The monosomic group had prolonged blastocyst parameters (tSB, tB, tB-tSB, P < 0.01), the trisomic group had prolonged t5-t2 (P < 0.05).

Deletion and duplication of different chromosomes have different effects on the morphokinetic parameters. Compared with euploid embryos, multiple morphokinetic parameters (from t4 to tB) significant delays in embryos only with partial or complete chromosome 20 deletion (from t4 to tB, P < 0.05, n = 8) or embryos only with partial or complete chromosome 22 deletion (from t4 to tSB, P < 0.05, n = 25).

## 4 Discussion

This research investigates the distinctive morphokinetic parameter features of euploid, mosaic, and aneuploid embryos. Additionally, it analyses the morphokinetic parameters characteristics of different types of aneuploidy and embryos with different abnormal chromosomal.

Due to differences in research design, patient characteristics, and statistical methods, there is significant heterogeneity among numerous studies exploring the relationship between morphokinetic parameters and embryo ploidy [14,15,22,[31], [32], [33], [34]]. In a recent meta-analysis, it was observed that certain variables, namely tPNa, t2, t4, and t7, exhibited less pronounced delays in aneuploid embryos compared to t8, t9, and tEB (the time from insemination to expanded blastocyst, when the blastocyst had increased in diameter by more than 30 % and the zona pellucida began to thin). Among the most delayed variables, t8 and t9 displayed minimal heterogeneity, with I^2^ values of 0 % and 33 %, respectively. However, tB and tEB exhibited substantial heterogeneity, with I^2^ values of 76 % and 83 %, respectively. tSB emerged as a statistically significant variable when analyzed using a fixed-effects model [14].

Most published studies overlook the influence of confounding variables, which can lead to model overestimation [14,35]. In previous studies, age has not been associated with morphokinetic parameters, and conclusions regarding the relationship between BMI and stimulus dose and morphokinetic parameters have been inconsistent [23,[36], [37], [38], [39], [40]]. In addition, the failure to account for the cluster effect is another shortcoming of most previous studies [21,23]. The changes in different TLM parameters are to some extent influenced by patient and cycle characteristics. Therefore, embryos originating from the same patient exhibit resemblances in their morphokinetic progression as opposed to embryos from different patients. Statistical tests such as the Mann-Whitney *U* test, Student t-test, analysis of variance, and ordinary logistic regression analysis operate under the assumption that each embryo is an independent object of observation. These data analysis methods, which do not consider the inherent clustering of embryos, may result in an overestimation of the reported impact [23]. Therefore, this study used a multilevel mixed-effects logistic regression model to adjust for confounding factors, considering the non-independence of different embryos from the same patient and the influence of patient- or cycle-related characteristics.

In confounding factor analysis, no specific patient or cycle characteristics were found to consistently influence the overall duration from cleavage to the blastocyst stage, which aligns with some findings of prior cluster analyses [23,31]. In Mumusoglu et al.'s study, it was observed that BMI exerted a significant influence on several parameters related to cleavage-stage morphokinetic timing, encompassing tPNa, tPNf, t2, t4, t5, t6, t7, and t8 [23]. The clustering analysis of Setti et al. show that serum AMH concentration was significantly negatively correlated with tPNf, t3, t4, t5, t6, t7, t8, and tB [40]. Our investigation, on the other hand, revealed that among all the TLM parameters examined, tB-tSB was the most susceptible to the influence of various patient- or cycle-related factors. After adjusting for confounding factors using a multi-level mixed effects logistic regression model, t5, t8, tSB, tB, t5-t2, tB-tSB were significant. In the study by Mumusoglu et al., the morphokinetic parameters of the blastocyst stage were significantly prolonged after adjusting for confounding factors [23].

Following the formulation of the Campbell model [41], there have been multiple endeavors to formulate and assess models employing morphokinetic parameters for predicting ploidy status [27,37,42]. However, replicating their outcomes in different laboratory settings has proven to be challenging [14]. Due to the wide range of time-lapse events, there were overlaps among euploid, mosaic, and aneuploid embryos in almost all time-lapse morphokinetic parameters, which makes it difficult to determine the threshold. Besides, these models did not adequately account for the impact of patient and ovarian stimulating factors and clustering. These limitations of the traditional model may be addressed through a combination of morphokinetic parameters and artificial intelligence. Artificial intelligence has showcased a remarkable capability in assessing pre-implantation embryo images, offering the advantage of conducting objective and swift analyses of an extensive volume of embryo videos. This capability proves beneficial in facilitating the advancement of large-scale multicenter research initiatives. Incorporating clinical characteristics into the algorithm for predicting ploidy based on morphokinetic parameters may enhance its predictive ability [43]. In a study conducted by Huang et al., it was noted that the Area Under the Curve (AUC) increased from 0.57 to 0.80 when patient age, blastocyst age (Day 5 or Day 6), and video data encompassing specific time intervals during both the cleavage and blastocyst stages were integrated into the analysis [44]. However, adding clinical characteristics to the model also introduces the risk that the algorithm might assign excessive weight to these covariates, resulting in minimal differentiation or ranking among embryos from the same patient. This effect is particularly problematic when selecting the best embryo for an individual patient, as factors like age may not provide useful insights at this level [45]. Despite the encouraging results, artificial intelligence models also have limitations. The morphokinetic behavior of embryos can be responsive to variations in laboratory conditions, and the utilization of different time-lapse equipment and embryo culture procedures may introduce variations in the morphokinetic parameters [35]. Differences in the way morphokinetic parameters are annotated and the degree of adherence to annotation practice guidelines [30] in different laboratories also reduce the generality of the model across laboratories. In addition, different laboratories may have different definitions of aneuploidy. As a result, current models may not translate across clinics or patient populations and require prospective multicenter large-sample studies for validation [46,47].

Different types of aneuploids have different effects on morphokinetic parameters [24,27]. In a previous study [27], embryos with trisomies showed very similar morphokinetic characteristics to euploids. Embryos with multiple chromosomal abnormalities (complexes) showed faster divisions than normal embryos significantly for t3, t5, cc2, cc3, s2, and t5-t2, and the embryos with monosomies showed intermediate morphokinetic characteristics between complex and euploids. In our study, morphokinetic parameters of late cleavage and blastocyst stages in embryos with chromosome fragment deletion or duplication were prolonged, and the morphokinetic parameters of the blastocyst stage in monosomic embryos were prolonged. Starting from four cells (t4), the development speed of fragment deletion embryos is significantly slower than that of euploid embryos. It should be noted that the tSB-t8 in the fragment deletion group was significantly shortened compared to the euploid group, possibly due to a more pronounced elongation of the cleavage stage than the initiation of blastulation. Figliuzzi et al. found that 60 % of fragment deletion embryos developed more slowly than their sibling euploid embryos from t4 [24]. In addition, they also found that trisomic embryos develop slowly after compaction completion (tM). However, we did not observe similar results in our study.

Gene imbalance caused by aneuploidy can lead to protein group imbalance, which may lead to the proliferation defect of aneuploid cells [[48], [49], [50], [51], [52]]. A recent study on the single-cell transcriptome atlas of human aneuploid and euploid blastocysts found that autosomal monosomy has a greater impact on the cell numbers of different blastocyst lineages and the abundance of chromosome transcripts compared to autosomal trisomy. Transcriptomic variations induced by aneuploidy may lead to substantial functional changes in aneuploid blastocysts, particularly monosomic blastocysts, thereby reducing their viability [53]. Previous studies have suggested that trisomic chromosomes seem to be subject to dosage compensation in blastocysts, which may in part be linked to chromosome-specific methylation profiles [17,54]. In our study, embryos with chromosomal segment deletions showed a more pronounced delay in morphokinetic parameters compared to those with chromosomal segment duplications, with this effect also significant in monosomy compared to trisomy. These findings suggest that the loss of chromosome copies, rather than an increase, is more likely to have a severe impact on embryo development. A recent study involving 99 mosaic embryos found that embryos with whole, segmental, and complex chromosomal mosaicism exhibited similar clinical outcomes [34]. Nevertheless, further research is needed to explore the differences in clinical outcomes among embryos with different types of chromosomal variations.

Aneuploid mouse cell lines carrying different extra chromosomes exhibit differences in gene expression, proliferation rate, cell volume, and metabolism [51]. Perhaps for these reasons, in our study, the deletion and duplication of different chromosomes have different effects on the morphokinetic parameters. The deletion or duplication of many chromosomes has no systematic effect on morphokinetic parameters. Compared with euploid embryos, multiple parameters of cleavage and blastocyst stages have significant delays in embryos only with partial or complete chromosome 20 or 22 deletion. This may imply that the abnormality of chromosomes 20 and 22 has an important influence on the proliferation of aneuploid cells. However, the relationship between aneuploidy and embryonic development is complex, and this conclusion needs to be verified by experiments or large-scale retrospective studies.

## 5 Conclusion

After adjusting for the patient- and cycle-related factors, prolonged morphokinetic parameters are common in aneuploid embryos, primarily during late cleavage and blastocyst stages. Certain types of chromosomal abnormalities, such as fragment deletions, fragment duplications, and monosomes, can lead to delays in late cleavage or blastocyst stages. The loss of chromosome copies may have a more severe impact on the morphokinetic characteristics of embryonic development compared to an increase. These findings provide insights into the development patterns of aneuploid embryos, aiding clinicians and embryologists in non-invasive embryo selection.

## CRediT authorship contribution statement

**Keyi Si:** Writing – original draft, Project administration, Methodology. **Bingxin Ma:** Validation, Software, Project administration. **Jian Bai:** Methodology. **Li Wu:** Supervision, Formal analysis, Data curation. **Hui He:** Methodology, Investigation, Formal analysis. **Lei Jin:** Supervision, Project administration, Investigation, Funding acquisition. **Bo Huang:** Supervision, Project administration, Conceptualization.

## Ethics approval and consent to participate

The study conformed to the Declaration of Helsinki for Medical Research Involving Human Subjects and was approved by the Ethics Committee of the Reproductive Medicine Center of Tongji Hospital. Informed consent was obtained from all individual participants included in the study.

## Consent for publication

Patients signed informed consent regarding publishing their data.

## Availability of data and materials

The datasets generated during and/or analyzed during the current study are available from the corresponding author on reasonable request.

## Funding

This work was supported by the 10.13039/501100012166National Key Research & Development Program of China (2021YFC2700603), China.

## Declaration of competing interest

The authors declare that they have no known competing financial interests or personal relationships that could have appeared to influence the work reported in this paper.

## List of abbreviations

(PGT): Preimplantation genetic testing
(TLM): Time-lapse microscopy
(ICSI): Intracytoplasmic Sperm Injection
(BMI): Body Mass Index
(FSH): Follicle-Stimulating Hormone
(AMH): Anti-Müllerian Hormone
(AUC): Area Under the Curve
(NGS): Next-Generation Sequencing

## References

[1] Fragouli E., Alfarawati S., Spath K., Jaroudi S., Sarasa J., Enciso M., Wells D. (2013). The origin and impact of embryonic aneuploidy. Hum. Genet..

[2] Templado C., Uroz L., Estop A. (2013). New insights on the origin and relevance of aneuploidy in human spermatozoa. Mol. Hum. Reprod..

[3] Viotti M. (2020). Preimplantation genetic testing for chromosomal abnormalities: aneuploidy, mosaicism, and structural rearrangements. Genes.

[4] Gleicher N., Patrizio P., Brivanlou A. (2021). Preimplantation genetic testing for aneuploidy - a castle built on sand. Trends Mol. Med..

[5] Kang H.-J., Melnick A.P., Stewart J.D., Xu K., Rosenwaks Z. (2016). Preimplantation genetic screening: who benefits?. Fertil. Steril..

[6] Practice Committee and Genetic Counseling Professional Group (GCPG) of the American Society for Reproductive Medicine (2020). Electronic address: asrm@asrm.org, Clinical management of mosaic results from preimplantation genetic testing for aneuploidy (PGT-A) of blastocysts: a committee opinion. Fertil. Steril..

[7] Yan J., Qin Y., Zhao H., Sun Y., Gong F., Li R., Sun X., Ling X., Li H., Hao C., Tan J., Yang J., Zhu Y., Liu F., Chen D., Wei D., Lu J., Ni T., Zhou W., Wu K., Gao Y., Shi Y., Lu Y., Zhang T., Wu W., Ma X., Ma H., Fu J., Zhang J., Meng Q., Zhang H., Legro R.S., Chen Z.-J. (2021). Live birth with or without preimplantation genetic testing for aneuploidy. N. Engl. J. Med..

[8] Apter S., Ebner T., Freour T., Guns Y., Kovacic B., Le Clef N., Marques M., Meseguer M., Montjean D., Sfontouris I., Sturmey R., Coticchio G., ESHRE Working group on Time-lapse technology (2020). Good practice recommendations for the use of time-lapse technology. Hum Reprod Open.

[9] Giménez C., Conversa L., Murria L., Meseguer M. (2023). Time-lapse imaging: morphokinetic analysis of in vitro fertilization outcomes. Fertil. Steril..

[10] Mio Y., Yumoto K., Sugishima M., Nakaoka M., Shimura T., Tsounapi P. (2024). Morphokinetic features in human embryos: analysis by our original high-resolution time-lapse cinematography-Summary of the past two decades. Reprod. Med. Biol..

[11] Cimadomo D., Marconetto A., Trio S., Chiappetta V., Innocenti F., Albricci L., Erlich I., Ben-Meir A., Har-Vardi I., Kantor B., Sakov A., Coticchio G., Borini A., Ubaldi F.M., Rienzi L. (2022). Human blastocyst spontaneous collapse is associated with worse morphological quality and higher degeneration and aneuploidy rates: a comprehensive analysis standardized through artificial intelligence. Hum. Reprod..

[12] Sciorio R., Campos G., Palini S., Baldini D., Janssens R. (2023). Real-time image and time-lapse technology to select the single blastocyst to transfer in assisted reproductive cycles. Zygote.

[13] Ezoe K., Takahashi T., Miki T., Kato K. (2024). Developmental perturbation in human embryos: clinical and biological significance learned from time-lapse images. Reprod. Med. Biol..

[14] Bamford T., Barrie A., Montgomery S., Dhillon-Smith R., Campbell A., Easter C., Coomarasamy A. (2022). Morphological and morphokinetic associations with aneuploidy: a systematic review and meta-analysis. Hum. Reprod. Update.

[15] Serrano-Novillo C., Uroz L., Márquez C. (2023). Novel time-lapse parameters correlate with embryo ploidy and suggest an improvement in non-invasive embryo selection. J. Clin. Med..

[16] Jin L., Si K., Li Z., He H., Wu L., Ma B., Ren X., Huang B. (2024). Multiple collapses of blastocysts after full blastocyst formation is an independent risk factor for aneuploidy - a study based on AI and manual validation. Reprod. Biol. Endocrinol..

[17] Hernandez Mora J.R., Buhigas C., Clark S., Del Gallego Bonilla R., Daskeviciute D., Monteagudo-Sánchez A., Poo-Llanillo M.E., Medrano J.V., Simón C., Meseguer M., Kelsey G., Monk D. (2023). Single-cell multi-omic analysis profiles defective genome activation and epigenetic reprogramming associated with human pre-implantation embryo arrest. Cell Rep..

[18] Coticchio G., Ezoe K., Lagalla C., Zacà C., Borini A., Kato K. (2023). The destinies of human embryos reaching blastocyst stage between Day 4 and Day 7 diverge as early as fertilization. Hum. Reprod..

[19] Kaing A., Kroener L.L., Tassin R., Li M., Liu L., Buyalos R., Hubert G., Shamonki M. (2018). Earlier day of blastocyst development is predictive of embryonic euploidy across all ages: essential data for physician decision-making and counseling patients. J. Assist. Reprod. Genet..

[20] Hernandez-Nieto C., Lee J.A., Slifkin R., Sandler B., Copperman A.B., Flisser E. (2019). What is the reproductive potential of day 7 euploid embryos?. Hum. Reprod..

[21] Kirkegaard K., Sundvall L., Erlandsen M., Hindkjær J.J., Knudsen U.B., Ingerslev H.J. (2016). Timing of human preimplantation embryonic development is confounded by embryo origin. Hum. Reprod..

[22] Chen C.-H., Lee C.-I., Huang C.-C., Chen H.-H., Ho S.-T., Cheng E.-H., Lin P.-Y., Chen C.-I., Lee T.-H., Lee M.-S. (2021). Blastocyst morphology based on uniform time-point assessments is correlated with mosaic levels in embryos. Front. Genet..

[23] Mumusoglu S., Yarali I., Bozdag G., Ozdemir P., Polat M., Sokmensuer L.K., Yarali H. (2017). Time-lapse morphokinetic assessment has low to moderate ability to predict euploidy when patient- and ovarian stimulation-related factors are taken into account with the use of clustered data analysis. Fertil. Steril..

[24] Figliuzzi M., Bori L., Caroselli S., Picchetta L., Reverenna M., Poli M., Campbell A., Smith R., Coticchio G., Cimadomo D., Rienzi L., Meseguer M., Capalbo A. (2023). Time-lapse imaging analysis of segmental aneuploid embryos: a multicenter study identifies morpho-kinetic patterns associated with chromosomal mosaicism. Hum. Reprod..

[25] Shahbazi M.N., Wang T., Tao X., Weatherbee B.A.T., Sun L., Zhan Y., Keller L., Smith G.D., Pellicer A., Scott R.T., Seli E., Zernicka-Goetz M. (2020). Developmental potential of aneuploid human embryos cultured beyond implantation. Nat. Commun..

[26] Zhu Y., Kratka C.R., Pea J., Lee H.C., Kratka C.E., Xu J., Marin D., Treff N.R., Duncan F.E. (2024). The severity of meiotic aneuploidy is associated with altered morphokinetic variables of mouse oocyte maturation. Hum Reprod Open.

[27] Del Carmen Nogales M., Bronet F., Basile N., Martínez E.M., Liñán A., Rodrigo L., Meseguer M. (2017). Type of chromosome abnormality affects embryo morphology dynamics. Fertil. Steril..

[28] Huang B., Tan W., Li Z., Jin L. (2021). An artificial intelligence model (euploid prediction algorithm) can predict embryo ploidy status based on time-lapse data. Reprod. Biol. Endocrinol..

[29] Gardner D.K., Lane M., Stevens J., Schlenker T., Schoolcraft W.B. (2000). Blastocyst score affects implantation and pregnancy outcome: towards a single blastocyst transfer. Fertil. Steril..

[30] Ciray H.N., Campbell A., Agerholm I.E., Aguilar J., Chamayou S., Esbert M., Sayed S. (2014). Time-Lapse User Group, Proposed guidelines on the nomenclature and annotation of dynamic human embryo monitoring by a time-lapse user group. Hum. Reprod..

[31] Kirkegaard K., Sundvall L., Erlandsen M., Hindkjær J.J., Knudsen U.B., Ingerslev H.J. (2016). Timing of human preimplantation embryonic development is confounded by embryo origin. Hum. Reprod..

[32] Quinn M.M., Marsh P., Ribeiro S., Simbulan R.K., Rosen M.P. (2022). A deep dive into the morphokinetics and ploidy of low-quality blastocysts. F S Rep.

[33] Figliuzzi M., Bori L., Caroselli S., Picchetta L., Reverenna M., Poli M., Campbell A., Smith R., Coticchio G., Cimadomo D., Rienzi L., Meseguer M., Capalbo A. (2023). O-077 Time-lapse imaging analysis of segmental aneuploid embryos: a multicenter study identifies morpho-kinetic patterns associated with chromosomal mosaicism. Hum. Reprod..

[34] Zou Y., Sui Y., Fu J., Ge N., Sun X., Sun Y. (2024). The morphokinetic signature of human blastocysts with mosaicism and the clinical outcomes following transfer of embryos with low-level mosaicism. J. Ovarian Res..

[35] Reignier A., Lammers J., Barriere P., Freour T. (2018). Can time-lapse parameters predict embryo ploidy? A systematic review. Reprod. Biomed. Online.

[36] Rienzi L., Capalbo A., Stoppa M., Romano S., Maggiulli R., Albricci L., Scarica C., Farcomeni A., Vajta G., Ubaldi F.M. (2015). No evidence of association between blastocyst aneuploidy and morphokinetic assessment in a selected population of poor-prognosis patients: a longitudinal cohort study. Reprod. Biomed. Online.

[37] Desai N., Goldberg J.M., Austin C., Falcone T. (2018). Are cleavage anomalies, multinucleation, or specific cell cycle kinetics observed with time-lapse imaging predictive of embryo developmental capacity or ploidy?. Fertil. Steril..

[38] Martín Á., Rodrigo L., Beltrán D., Meseguer M., Rubio C., Mercader A., de Los Santos M.J. (2021). The morphokinetic signature of mosaic embryos: evidence in support of their own genetic identity. Fertil. Steril..

[39] Ezoe K., Miki T., Akaike H., Shimazaki K., Takahashi T., Tanimura Y., Amagai A., Sawado A., Mogi M., Kaneko S., Ueno S., Coticchio G., Cimadomo D., Borini A., Rienzi L., Kato K. (2023). Maternal age affects pronuclear and chromatin dynamics, morula compaction and cell polarity, and blastulation of human embryos. Hum. Reprod..

[40] Setti A.S., Braga D.P. de A.F., Guilherme P., Iaconelli A., Borges E. (2023). Serum anti-Müllerian hormone concentrations are related to embryo development: lessons from time-lapse imaging. Zygote.

[41] Campbell A., Fishel S., Bowman N., Duffy S., Sedler M., Hickman C.F.L. (2013). Modelling a risk classification of aneuploidy in human embryos using non-invasive morphokinetics. Reprod. Biomed. Online.

[42] Basile N., Nogales M. del C., Bronet F., Florensa M., Riqueiros M., Rodrigo L., García-Velasco J., Meseguer M. (2014). Increasing the probability of selecting chromosomally normal embryos by time-lapse morphokinetics analysis. Fertil. Steril..

[43] Zou Y., Pan Y., Ge N., Xu Y., Gu R., Li Z., Fu J., Gao J., Sun X., Sun Y. (2022). Can the combination of time-lapse parameters and clinical features predict embryonic ploidy status or implantation?. Reprod. Biomed. Online.

[44] Huang B., Tan W., Li Z., Jin L. (2021). An artificial intelligence model (euploid prediction algorithm) can predict embryo ploidy status based on time-lapse data. Reprod. Biol. Endocrinol..

[45] A C., A C., B. T, S. R, E. C, D.-S. R, B. A, M. S (2023). Association between a morphokinetic ploidy prediction model risk score and miscarriage and live birth: a multicentre cohort study. Fertil. Steril..

[46] Li X., Yao Y., Zhao D., Chang X., Li Y., Lin H., Wei H., Wang H., Mi Y., Huang L., Lu S., Yang W., Cai L. (2024). Clinical outcomes of single blastocyst transfer with machine learning guided noninvasive chromosome screening grading system in infertile patients. Reprod. Biol. Endocrinol..

[47] Riegler M.A., Stensen M.H., Witczak O., Andersen J.M., Hicks S.A., Hammer H.L., Delbarre E., Halvorsen P., Yazidi A., Holst N., Haugen T.B. (2021). Artificial intelligence in the fertility clinic: status, pitfalls and possibilities. Hum. Reprod..

[48] Oromendia A.B., Amon A. (2014). Aneuploidy: implications for protein homeostasis and disease. Dis Model Mech.

[49] Singla S., Iwamoto-Stohl L.K., Zhu M., Zernicka-Goetz M. (2020). Autophagy-mediated apoptosis eliminates aneuploid cells in a mouse model of chromosome mosaicism. Nat. Commun..

[50] Torres E.M., Sokolsky T., Tucker C.M., Chan L.Y., Boselli M., Dunham M.J., Amon A. (2007). Effects of aneuploidy on cellular physiology and cell division in haploid yeast. Science.

[51] Williams B.R., Prabhu V.R., Hunter K.E., Glazier C.M., Whittaker C.A., Housman D.E., Amon A. (2008). Aneuploidy affects proliferation and spontaneous immortalization in mammalian cells. Science.

[52] Licciardi F., Lhakhang T., Kramer Y.G., Zhang Y., Heguy A., Tsirigos A. (2018). Human blastocysts of normal and abnormal karyotypes display distinct transcriptome profiles. Sci. Rep..

[53] Wang S., Leng L., Wang Q., Gu Y., Li J., An Y., Deng Q., Xie P., Cheng C., Chen X., Zhou Q., Lu J., Chen F., Liu L., Yang H., Wang J., Xu X., Hou Y., Gong F., Hu L., Lu G., Shang Z., Lin G. (2024). A single-cell transcriptome atlas of human euploid and aneuploid blastocysts. Nat. Genet..

[54] Yang M., Tao X., Scott K., Zhan Y., Scott R.T., Seli E. (2021). Evaluation of genome-wide DNA methylation profile of human embryos with different developmental competences. Hum. Reprod..

