# Supplementary material for: Preimplantation development analysis of aneuploid embryos with different chromosomal abnormalities
Source: Heliyon. 2024 Nov 26;10(23):e40686. doi: 10.1016/j.heliyon.2024.e40686 (PMC11647804; doi:10.1016/j.heliyon.2024.e40686)
Supplement: Multimedia component 1 [file mmc1.docx]

**Supplement Table I.** Comparison of morphokinetic parameters in blastocysts with different ploidy statuses.

| Morphokinetic  parameters | Euploid  (n=1260) | Mosaic  (n=331) | Aneuploid  (n=1285) |
| --- | --- | --- | --- |
| tPNa (hpi) (n=2842) | 8.1±2.3 | 8.1±2.1 | 8.1±2.2 |
| tPNf (hpi) (n=2846) | 22.7±2.9 | 22.5±2.7 | 22.6±2.7 |
| t2 (hpi) (n=2852) | 25.3±3 | 25.3±2.8 | 25.3±2.8 |
| t3 (hpi) (n=2744) | 35.6±4.4 | 35.4±4.5 | 35.7±4 |
| t4 (hpi) (n=2815) | 37.0±4.3 | 37.1±4.2 | 37.1±4.1 |
| t5 (hpi) (n=2759) | 48.0±6.9 | 48.5±7.9 | 48.7±6.6 |
| t6 (hpi) (n=2534) | 50.7±6.2 | 51.3±7.2 | 51.0±6.1 |
| t7 (hpi) (n=2520) | 52.8±6.7 | 53.6±8.1 | 53.1±6.7 |
| t8 (hpi) (n=2605) | 55.7±8.2 | 57.0±9.7 | 56.3±8.5 |
| tSB (hpi) (n=2713) | 97.8±8 | 98.3±8.8 | 98.9±8.2 |
| tB (hpi) (n=2605) | 107.9±8.8 | 108.4±9.1 | 110.2±8.9 |
| tPNf-tPNa (h) (n=2830) | 14.6±2.8 | 14.4±2.9 | 14.5±2.8 |
| t2-tPNf (h) (n=2833) | 2.6±0.6 | 2.7±0.7 | 2.6±0.6 |
| t5-t2 (h) (n=2742) | 22.7±5.7 | 23.2±6.7 | 23.4±5.5 |
| tSB-t8 (h) (n=2474) | 41.8±8.4 | 41.1±10.2 | 42.5±8.9 |
| tB-tSB (h) (n=2695) | 10.2±3.8 | 10.2±3.9 | 11.3±4.4 |
| ECC2 (h) (n=2800) | 11.6±2.5 | 11.8±2.7 | 11.7±2.4 |
| ECC3 (h) (n=2585) | 18.8±6.8 | 19.8±7.9 | 19.3±6.9 |
| s2 (h) (n=2719) | 1.4±2.7 | 1.6±2.9 | 1.3±2.3 |
| s3 (h) (n=2567) | 7.7±7.3 | 8.4±7.8 | 7.6±7.0 |
