# Supplementary material for: Preimplantation development analysis of aneuploid embryos with different chromosomal abnormalities
Source: Heliyon. 2024 Nov 26;10(23):e40686. doi: 10.1016/j.heliyon.2024.e40686 (PMC11647804; doi:10.1016/j.heliyon.2024.e40686)
Supplement: Multimedia component 2 [file mmc2.docx]

**Supplement Table 2.**

Multilevel mixed-effects linear regression model analysis for confounding effects and time-lapse morphokinetic parameters.

| TLM parameters | Female age (y) | BMI (kg/m^2^) | Duration of infertility (y) | Number of retrieved oocytes | Level of FSH/ 100 (IU) | Level of AMH/ 100 (IU) | Time of ovarian stimulation (days) |
| --- | --- | --- | --- | --- | --- | --- | --- |
| tPNa (hpi) (n=2842) | 0.004 (-0.023 to 0.030) | -0.014 (-0.054 to 0.025) | -0.005 (-0.057 to 0.047) | -0.002 (-0.023 to 0.014) | 0.005 (-0.036 to 0.045) | -0.030 (-0.065 to 0.005) | -0.014 (-0.080 to 0.052) |
| tPNf (hpi) (n=2846) | 0.003 (-0.037 to 0.043) | -0.023 (-0.075 to 0.028) | 0.058 (-0.011 to 0.126) | -0.015 (-0.042 to 0.011) | -0.026 (-0.079 to 0.027) | -0.020 (-0.079 to 0.039) | -0.046 (-0.140 to 0.037) |
| t2 (hpi) (n=2852) | -0.001 (-0.042 to 0.040) | -0.013 (-0.068 to 0.041) | 0.064 (-0.006 to 0.134) | -0.014 (-0.042 to 0.015) | -0.030 (-0.086 to 0.027) | -0.019 (-0.081 to 0.042) | -0.045 (-0.134 to 0.044) |
| t3 (hpi) (n=2744) | 0.007 (-0.046 to 0.060) | -0.009 (-0.082 to 0.064) | 0.037 (-0.079 to 0.152) | -0.026 (-0.066 to 0.014) | -0.030 (-0.113 to 0.053) | 0.018 (-0.066 to 0.014) | >-0.001 (-0.117 to 0.117) |
| t4 (hpi) (n=2815) | 0.003 (-0.052 to 0.058) | -0.048 (-0.121 to 0.026) | 0.085 (-0.013 to 0.182) | -0.017 (-0.058 to 0.024) | -0.004 (-0.079 to 0.070) | 0.029 (-0.060 to 0.117) | -0.029 (-0.151 to 0.093) |
| t5 (hpi) (n=2759) | 0.029 (-0.054 to 0.112) | -0.006 (-0.125 to 0.112) | 0.029 (-0.131 to 0.190) | -0.003 (-0.063 to 0.056) | 0.019 (-0.107 to 0.144) | 0.038 (-0.091 to 0.166) | 0.058 (-0.114 to 0.230) |
| t6 (hpi) (n=2534) | 0.027 (-0.053 to 0.108) | -0.034 (-0.145 to 0.078) | 0.112 (-0.021 to 0.245) | -0.022 (-0.079 to 0.036) | 0.046 (-0.059 to 0.152) | 0.085 (-0.044 to 0.214) | 0.056 (-0.109 to 0.220) |
| t7 (hpi) (n=2520) | -0.004 (-0.087 to 0.079) | 0.022 (-0.110 to 0.154) | 0.101 (-0.050 to 0.253) | -0.023 (-0.085 to 0.039) | 0.024 (-0.110 to 0.157) | 0.049 (-0.090 to 0.189) | 0.068 (-0.128 to 0.263) |
| t8 (hpi) (n=2605) | 0.059 (-0.048 to 0.167) | -0.005 (-0.171 to 0.162) | 0.123 (-0.071 to 0.317) | -0.009 (-0.081 to 0.064) | 0.114 (-0.077 to 0.305) | 0.061 (-0.112 to 0.235) | 0.148 (-0.107 to 0.402) |
| tSB (hpi) (n=2713) | 0.069 (-0.033 to 0.172) | -0.021 (-0.167 to 0.126) | -0.016 (-0.182 to 0.150) | -0.066 (-0.136 to 0.004) | 0.106 (-0.050 to 0.262) | -0.039 (-0.178 to 0.101) | 0.252 (0.008 to 0.496)* |
| tB (hpi) (n=2605) | 0.024 (-0.092 to 0.139) | 0.072 (-0.080 to 0.225) | 0.087 (-0.100 to 0.274) | -0.074 (-0.150 to 0.001) | 0.033 (-0.134 to 0.200) | -0.005 (-0.158 to 0.148) | 0.440 (0.159 to 0.720)* |
| tPNf-tPNa (h) (n=2830) | <0.001 (-0.038 to 0.038) | -0.010 (-0.056 to 0.036) | 0.061 (-0.008 to 0.130) | -0.015 (-0.039 to 0.009) | -0.028 (-0.077 to 0.022) | 0.017 (-0.036 to 0.070) | -0.035 (-0.119 to 0.049) |
| t2-tPNf (h) (n=2833) | 0.004 (-0.003 to 0.010) | 0.010 (0.001 to 0.019)* | 0.002 (-0.012 to 0.016) | 0.003 (-0.003 to 0.008) | 0.001 (-0.009 to 0.010) | -0.001 (-0.012 to 0.011) | -0.007 (-0.023 to 0.009) |
| t5-t2 (h) (n=2742) | 0.031 (-0.033 to 0.095) | -0.002 (-0.094 to 0.089) | -0.040 (-0.165 to 0.085) | 0.006 (-0.034 to 0.046) | 0.039 (-0.060 to 0.138) | 0.053 (-0.033 to 0.140) | 0.093 (-0.037 to 0.223) |
| tSB-t8 (h) (n=2474) | 0.005 (-0.107 to 0.118) | -0.002 (-0.169 to 0.165) | -0.073 (-0.271 to 0.124) | -0.035 (-0.098 to -0.029) | -0.069 (-0.234 to 0.097) | -0.125 (-0.276 to 0.026) | 0.172 (-0.111 to 0.454) |
| tB-tSB (h) (n=2695) | -0.050 (-0.102 to 0.001) | 0.085 (0.026 to 0.143)** | 0.109(0.021 to 0.197)* | -0.011 (-0.037 to 0.016) | -0.067 (-0.131 to -0.002)* | 0.040 (-0.018 to 0.098) | 0.173 (0.062 to 0.284)** |
| ECC2 (h) (n=2800) | 0.011 (-0.015 to 0.036) | -0.028 (-0.060 to 0.004) | <0.001 (-0.039 to 0.040) | 0.001 (-0.017 to 0.019) | 0.021 (-0.017 to 0.059) | 0.016 (-0.022 to 0.053) | 0.029 (-0.024 to 0.081) |
| ECC3 (h) (n=2585) | 0.023 (-0.059 to 0.105) | 0.020 (-0.115 to 0.155) | 0.061 (-0.106 to 0.229) | -0.004 (-0.055 to 0.046) | 0.151 (0.002 to 0.229)* | 0.033 (-0.091 to 0.157) | 0.126 (-0.065 to 0.317) |
| s2 (h) (n=2719) | -0.004 (-0.026 to 0.019) | -0.011 (-0.042 to 0.019) | 0.012 (-0.044 to 0.069) | 0.008 (-0.007 to 0.023) | 0.015 (-0.023 to 0.054) | -0.005 (-0.035 to 0.026) | -0.041 (-0.091 to 0.009) |
| s3 (h) (n=2567) | 0.021 (-0.057 to 0.098) | -0.009 (-0.119 to 0.102) | 0.128 (-0.036 to 0.292) | <0.001 (-0.050 to 0.049) | 0.125 (-0.015 to 0.265) | 0.019 (-0.100 to 0.137) | 0.048 (-0.149 to 0.245) |

The groups with significant differences (P<0.05) were marked. (*, P<0.05; **, P<0.01; ***, P<0.001). BMI, Body Mass Index; FSH, Follicle-Stimulating Hormone; AMH, Anti-Müllerian Hormone.
