# Supplementary material for: Preimplantation development analysis of aneuploid embryos with different chromosomal abnormalities
Source: Heliyon. 2024 Nov 26;10(23):e40686. doi: 10.1016/j.heliyon.2024.e40686 (PMC11647804; doi:10.1016/j.heliyon.2024.e40686)
Supplement: Multimedia component 3 [file mmc3.docx]

**Supplement Table 3.** Comparison of morphokinetic parameters in blastocysts with different chromosome abnormalities.

| Morphokinetic  parameters | Euploid  (n=1260) | Fragmental deletion  (n=130) | Fragmental duplication  (n=114) | Monosomic  (n=131) | Trisomic  (n=96) |
| --- | --- | --- | --- | --- | --- |
| tPNa (hpi) (n=1714) | 8.1±2.3 | 8.2±2.3 | 8.1±1.9 | 7.8±2.1 | 7.8±2.0 |
| tPNf (hpi)  (n=1713) | 22.7±2.9 | 22.9±2.5 | 22.9±2.7 | 22.6±2.8 | 22.6±2.8 |
| t2 (hpi) (n=1718) | 25.3±3.0 | 25.5±2.7 | 25.5±2.7 | 25.3±2.8 | 25.2±2.8 |
| t3 (hpi) (n=1650) | 35.6±4.4 | 35.8±4.1 | 36.1±3.6 | 35.7±3.9 | 36.0±3.5 |
| t4 (hpi) (n=1699) | 37.0±4.3 | 37.7±3.8 | 37.4±3.5 | 36.8±3.6 | 36.7±3.6 |
| t5 (hpi) (n=1662) | 48.0±6.9 | 49.3±7.1 | 49.6±6.4 | 48.7±6.7 | 49.0±6.5 |
| t6 (hpi) (n=1541) | 50.7±6.2 | 52.0±6.2 | 51.2±5.9 | 51.4±6.7 | 51.0±6.1 |
| t7 (hpi) (n=1525) | 52.8±6.7 | 55.0±7.2 | 53.8±6.1 | 54.1±8.0 | 52.5±6.1 |
| t8 (hpi) (n=1576) | 55.7±8.2 | 58.2±9.2 | 56.7±8.6 | 57.1±8.9 | 56.4±8.0 |
| tSB (hpi) (n=1643) | 97.8±8.0 | 98.6±8.5 | 99.3±7.7 | 100.1±8.0 | 98.1±8.1 |
| tB (hpi) (n=1637) | 107.9±8.8 | 110.5±8.5 | 109.9±9.2 | 111.4±8.8 | 108.6±8.9 |
| tPNf-tPNa (h) (n=1706) | 14.6±2.8 | 14.7±2.7 | 14.8±2.4 | 14.8±2.7 | 14.8±2.7 |
| t2-tPNf (h) (n=1708) | 2.6±0.6 | 2.6±0.8 | 2.6±0.4 | 2.6±0.4 | 2.6±0.3 |
| t5-t2 (h) (n=1651) | 22.7±5.7 | 23.6±6.2 | 24.1±5.6 | 23.4±5.7 | 23.8±5.5 |
| tSB-t8 (h) (n=1506) | 41.8±8.4 | 40.1±9.0 | 42.5±8.0 | 43.0±8.1 | 40.7±9.3 |
| tB-tSB (h) (n=1633) | 10.2±3.8 | 11.9±3.9 | 10.6±3.9 | 11.3±4.3 | 10.6±4.3 |
| ECC2 (h) (n=1690) | 11.6±2.5 | 12.2±2.5 | 11.9±1.8 | 11.5±2.0 | 11.6±2.3 |
| ECC3 (h) (n=1566) | 18.8±6.8 | 20.5±7.9 | 19.4±7.2 | 20.1±7.6 | 19.7±6.7 |
| s2 (h) (n=1636) | 1.4±2.7 | 1.8±2.7 | 1.2±2.1 | 1.1±2.1 | 0.9±1.1 |
| s3 (h) (n=1577) | 7.7±7.3 | 9.0±8.0 | 7.0±7.2 | 8.3±7.4 | 7.8±7.0 |
